# Medical treatment of orthotopic glioblastoma with transferrin-conjugated nanoparticles encapsulating zoledronic acid

**DOI:** 10.18632/oncotarget.2182

**Published:** 2014-07-09

**Authors:** Manuela Porru, Silvia Zappavigna, Giuseppina Salzano, Amalia Luce, Antonella Stoppacciaro, Maria Luisa Balestrieri, Simona Artuso, Sara Lusa, Giuseppe De Rosa, Carlo Leonetti, Michele Caraglia

**Affiliations:** ^1^ Experimental Chemotherapy Laboratory, Regina Elena National Cancer Institute, Via delle Messi d'oro, Rome, Italy; ^2^ Department of Biochemistry, Biophysics and General Pathology, Second University of Naples, Via Costantinopoli, Naples, Italy; ^3^ Department of Pharmacy, University of Naples Federico II, Via Montesano, Naples, Italy

**Keywords:** Glioblastoma, intracranial xenografts, zoledronic acid, transferrin, delivery, calcium phosphate self-assembly nanoparticles

## Abstract

Glioblastomas are highly aggressive adult brain tumors with poor clinical outcome. In the central nervous system (CNS) the blood-brain barrier (BBB) is the most important limiting factor for both development of new drugs and drug delivery. Here, we propose a new strategy to treat glioblastoma based on transferrin (Tf)-targeted self-assembled nanoparticles (NPs) incorporating zoledronic acid (ZOL) (NPs-ZOL-Tf). NPs-ZOL-Tf have been assessed on the glioblastoma cell line U373MG-LUC that showed a refractoriness *in vitro* to temozolomide (TMZ) and fotemustine (FTM). NPs-ZOL-Tf treatment resulted in higher in vitro cytotoxic activity than free ZOL. However, the potentiation of anti-proliferative activity of NPs-ZOL-Tf was superimposable to that one induced by NPs-ZOL (not armed with Tf). On the other hand, NPs-ZOL-Tf showed a higher antitumor efficacy if compared with that one caused by NPs-ZOL in immunosuppressed mice intramuscularly bearing U373MG-LUC xenografts, inducing a significant tumor weight inhibition (TWI). The experiments performed on mice with intracranial U373MG-LUC xenografts confirmed the efficacy of NPs-ZOL-Tf. These effects were paralleled by a higher intratumour localization of fluorescently-labeled-NPs-Tf both in intramuscular and intracranial xenografts. In conclusion, our results demonstrate that the encapsulation of ZOL increases the antitumor efficacy of this drug in glioblastoma through the acquisition of ability to cross the BBB.

## INTRODUCTION

Glioblastoma [glioblastoma multiforme (GBM)] is the most common and aggressive type of adult primary central nervous system tumor. Median survival of patients with GBM is up-to 40–50 weeks when treated with surgery, radiotherapy and chemotherapy. The gold-standard first-line treatment for GBM is based on a combination of radiotherapy and temozolomide (TMZ), as derived from the study by Stupp et al [1] on a total of 573 randomized patients. An improved survival was largely restricted to a subset of patients harboring promoter methylation of DNA repair gene O6-methylguanine-DNA methyltransferase [2]. Despite the aggressive first-line therapy, tumors invariably recur and median survival is 15-18 months and less than 10% of patients are alive at 5 years. TMZ is the best-documented agent and has shown a single-agent response rate of 5–8% in GBM, and of 35% in anaplastic astrocytoma [3]. Recent phase II trials have demonstrated activity of nitrosoureas such as fotemustine (FTM) in recurrent GBM [4, 5]. FTM is a third-generation nitrosourea with an alkylating cytotoxic activity, characterized by a phosphoalanine carrier group grafted onto the nitrosourea radical, which gives it high lipophilicity that allows it to cross the blood–brain barrier (BBB) [6, 7]. FTM showed both *in vitro* and *in vivo* marked antineoplastic activity on human GBM and medulloblastoma cell lines [8].

Despite a broader range of new and more specific treatment strategies, therapy of GBM remains challenging.

The results found by our group by using zoledronic acid (ZOL) in different experimental models of solid and haematological tumors [9-13] encouraged us to investigate additional potentialities of this drug, for example against GBM. ZOL is the most potent aminobisphosphonate, able to induce cell growth inhibition and apoptosis at very low concentrations [14]. Many authors hypothesize that a direct anti-tumor activity should be responsible for the positive effect of ZOL on bone metastases. The lack of clear evidence of ZOL-induced anti-cancer effects is likely due to its unfavourable pharmacokinetic profile. In fact, it accumulates almost exclusively in the bone and has a short serum half-life (only 15 min) not reaching active anti-tumor concentrations [15]. Our group demonstrated that the use of nanotechnology-based formulations overcomes these limitations due to bad ZOL pharmaco-distribution changing this drug in a powerful anticancer agent [9, 16].

On the other hand, nanotechnologies have been proposed to enhance drug delivery into the CNS [17]. Indeed, drug transport from the bloodstream to the CNS is hindered by the presence of an endothelium characterized by a low permeability, namely the BBB, whose cells are linked by tight junctions hindering the passage of most drugs [18, 19]. Nanocarriers can access to the brain by exploiting the enhanced BBB permeability typical of some intracranial tumors (above all metastases) [17, 20]. Moreover, mechanism of receptor-mediated transcytosis (RMT) can be used by binding different ligands, such as transferrin (Tf), to the nanocarrier surface in order to enhance crossing of the BBB [21]. On the basis of all these considerations, we proposed a previously developed delivery system consisting in self-assembling PEGylated nanoparticles (NPs) [22], based on calcium/phosphate NPs and cationic liposomes, to deliver ZOL in GBM cells. However, to improve the delivery of ZOL into the brain, we developed ZOL-containing NPs (NPs-ZOL) functionalized with Tf able to bind specific receptors on endothelial cells of BBB. Moreover, the overexpression of Tf receptor on the surface of GBM cells could allow using these newly developed NPs to actively target GBM cells. The Tf insertion on the NP surface was carried out maintaining the self-assembling characteristics of the NPs; consequently, these NPs can be prepared immediately before use in hospital setting, thus avoiding storage concerns. These features of the developed NPs should facilitate their scale-up process and their subsequent commercial development. In the present manuscript, we have evaluated the antitumor effects of NPs-ZOL functionalized or not with transferrin on intramuscularly and intracranially human GBM xenografts.

## RESULTS

### ZOL-containing self-assembling NPs

The developed self-assembling NPs had a mean diameter of about 147 nm with a very narrow size distribution (PI< 0.2) and an actual loading of ~100 μg of ZOL/mg lipids. The use of Tf in the preparation procedure, did not significantly affected the NP characteristics in terms of size and ZOL encapsulation.

### *In vitro* effects of NPs-ZOL formulations, TMZ and FTM on GBM cells

In order to evaluate the *in vitro* cytotoxic effects of different NPs-ZOL formulations in comparison to the standard antineoplastic drugs, TMZ and FTM, on U373MG-LUC GBM cells, we treated the cells with increasing concentrations of free ZOL, NPs-ZOL, NPs-ZOL-Tf, TMZ and FTM for 72h. Cell proliferation was assessed by MTT assay and cell survival by clonogenic assay, as described in “Materials and Methods”.

**Figure 1:**
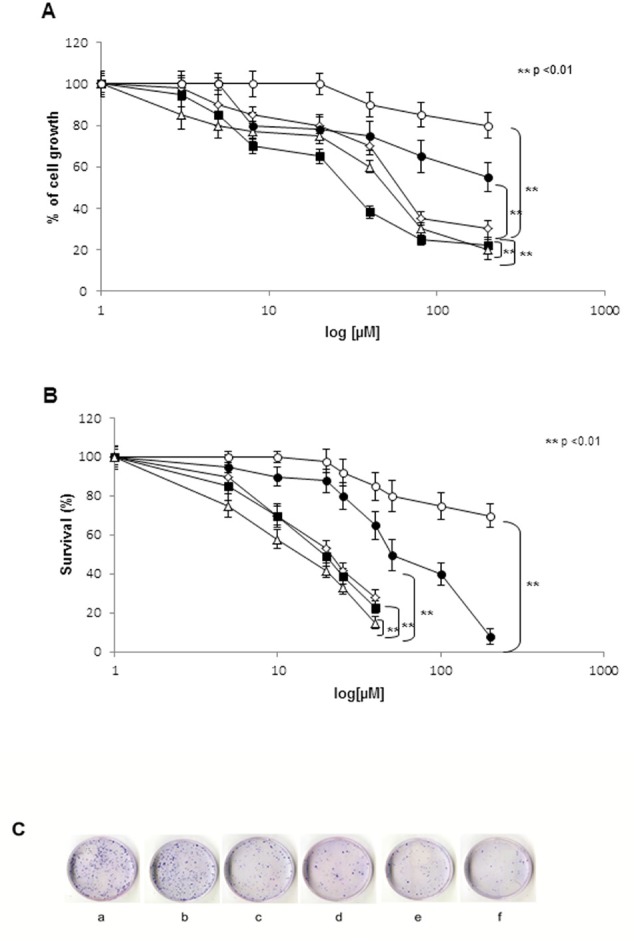
NPs- ZOL-Tf have cytotoxic activity on U373 MG- LUC cells A). U373 MG- LUC GBM cells (2×10^3^) were seeded in 96-well plates in serum-containing media and allowed to attach for 24 hrs. The medium was then removed and replaced with new medium containing TMZ (●), FTM (○), ZOL (◊), NPs-ZOL (■) and NPs-ZOL-Tf (Δ) at different concentrations. Cells were incubated under these conditions for a time course spanning 72 hrs. Then cell viability was assessed with MTT method. The MTT solution (5.0 mg/mL in phosphate-buffered saline) was added (20 μL/well), and the plates were incubated for further 4 hrs at 37° C. The purple formazan crystals were dissolved in 200 μL isopropanol per well. After the plates were read on microplate reader at 540 nm. The figure shows representative experiments performed in triplicate with SDs. B). U373 MG- LUC cells were seeded in 60 mm- Petri dishes at a density of 5 x10^4^ cells/plate in RPMI medium. After 24 hours, cells were treated with TMZ (●) FTM (○), ZOL (◊), NPs-ZOL (■) or NPs-ZOL-Tf (Δ) for 72 hrs at different concentrations. To evaluate cell colony- forming ability, at the end of treatments cells were trypsinized, counted and aliquots from each sample were seeded in triplicate in 60 mm dishes into complete medium at a density of 500 cells. After 10 days colonies were stained with 2% methylene blue in 95% of ethanol and counted (> 50 cells equaled one colony). Data are means ± SD of 3 independent experiments. The survival of cells treated with NPs-ZOL-Tf was significantly decreased (P< 0.01) compared to untreated and to all treated groups. C) Representative images of clonogenic ability of the untreated (a), FTM (b), TMZ (c), ZOL (d), NPs-ZOL (e) and NPs-ZOL-Tf (f) were showed.

After 72h of treatment, we showed that the encapsulation in NPs increased the efficacy of ZOL. In fact, the analysis of U373MG-LUC growth in terms of MTT assay (Figure 1A) demonstrated that free ZOL induced the 50% growth inhibition at a concentration of 46 μM (IC_50_) (Table 1). This effect was enhanced when the ZOL was encapsulated into NPs; in fact, NPs-ZOL showed an IC_50_ equal to 20 μM in U373MG-LUC,that is more than half compared to the free drug. In the other hand, NPs-ZOL-Tf showed a decrease of IC_50_ compared to free ZOL but not respect to NPs-ZOL (IC_50_ 30 μM) (Table 1).

**Table I:** Therapeutic efficacy of NPs- ZOL-TfU373MG-LUC on glioblastoma heterotopic xenografts

Treatments	Cell Growth IC_50_	Survival IC_50_
a) FTM	>600 μM	> 400 μM
b) TMZ	110 μM	56 μM
c) ZOL	46 μM	21 μM
d) NPs-ZOL	20μM	18 μM
e) NPs-ZOL- Tf	30 μM	12.5 μM

IC_50_ values of different treatments against U373-MG-LUC glioblastoma cells.

U373-MG-LUC glioblastoma cells were treated with the compounds as reported in materials and methods and IC50 calculated both in terms of cell growth and colony formation.

Statistical analysis of cell growth: NP-ZOL vs freeZOL, p≤0.001; NP-ZOL-Tf vs free ZOL, p≤0.001; FTM vs ZOL p≤0.001; and TMZ vs ZOL p≤0.001

Statistical analysis of colony formation: b vs untreated, p= 0.0012; c vs untreated, p= 0.00004, d vs untreated, p=0.00002, e vs untreated p= 0.00001; b vs c, p= 0.00007; b vs d, p= 0.00003; e vs b, p= 0.00001; e vs c, p= 0.0007; e vs d, p= 0.0004.

Statistical comparison with FTM (a) was not possible since IC50 was not reached.

Anti-cancer agents conventionally used in the clinic for the treatment of GBM are TMZ and FTM. In particular, TMZ is currently used in combination with radiation therapy or alone after surgery and radiotherapy [1, 2]. On these bases, we evaluated the cytotoxic effects of the TMZ and FTM on this GBM cell line. After 72h the IC_50_ of TMZ was 110 μM for U373MG-LUC and this line was highly resistant to the treatment with FTM with an IC_50_ of more than 600 μM, thus suggesting an *in vitro* refractoriness of human GBM cells both to TMZ and FTM (Table 1).

These observations were confirmed when the efficacy of different formulations was analyzed in terms of clonogenic assay (Figure 1B). In fact, the IC_50_ of free ZOL was 21 μM and decreased to 18 μM for NPs-ZOL and to 12 for μM NPs-ZOL-Tf. Also in the clonogenic assay, U373MG-LUC cells exhibited low sensitivity to TMZ and a strong resistance to FTM.

In all cases, empty NPs did not reduce growth and survival of cells if not at high concentrations demonstrating a very low cytotoxicity (data not shown).

### Antitumor effect of NPs-ZOL formulations against heterotopic GBM xenografts

To study the *in vivo* effects of NP formulations, we inoculated i.m. 3 × 10^6^ U373MG-LUC cells into immunosuppressed mice. U-373MG-LUC cells allow the *in vivo* monitoring of tumor growth by imaging performed as a function of the bioluminescent signal generated by the catalysis of D-luciferin, injected in the animal at the time of imaging.

**Figure 2:**
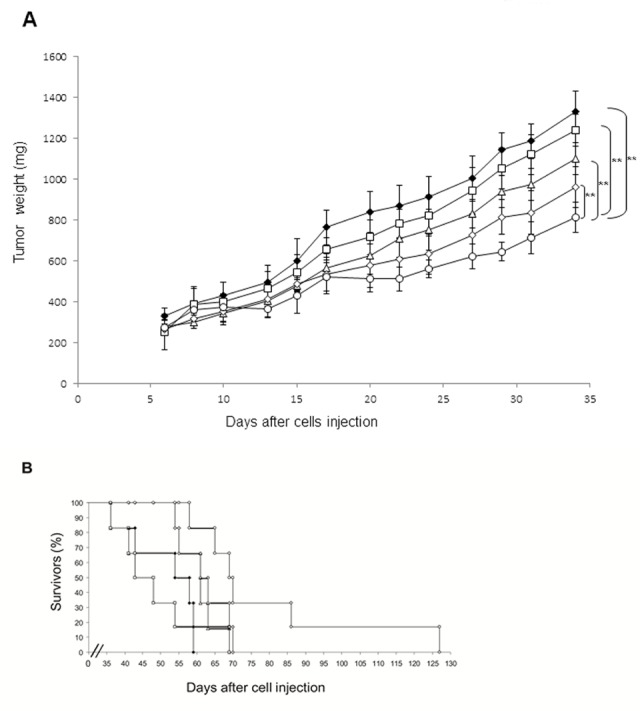
NPs-ZOL-Tf riduce the growth of U373 MG-LUC tumors in heterotopic xenografts Immunosuppressed mice were injected intramuscularly (i.m.) into the hind leg muscles of mice at 3×10^6^ U373 MG-LUC GBM cells /mouse. After 6 days (when a tumor mass of about 300 mg was evident) mice were randomized, divided in five groups and treatment started. The following groups were evaluated: (♦) control; (€) free ZOL at 20 μg/mouse; (Δ) NPs- Tf; (◊) NPs-ZOL at 20 μg/mouse and (○) NPs- ZOL-Tf at 20 μg/mouse. Mice were treated intravenously (i.v.) for three times a week for 3 consecutive weeks. A) Tumor sizes were measured three times a week in two dimensions by a caliper and tumor weight was calculated using the following formula: a×b^2^/2, where a and b are the long and short diameter of the tumor, respectively. Error bars indicate ±SD. B) Survival curves are reported.

**Figure 3:**
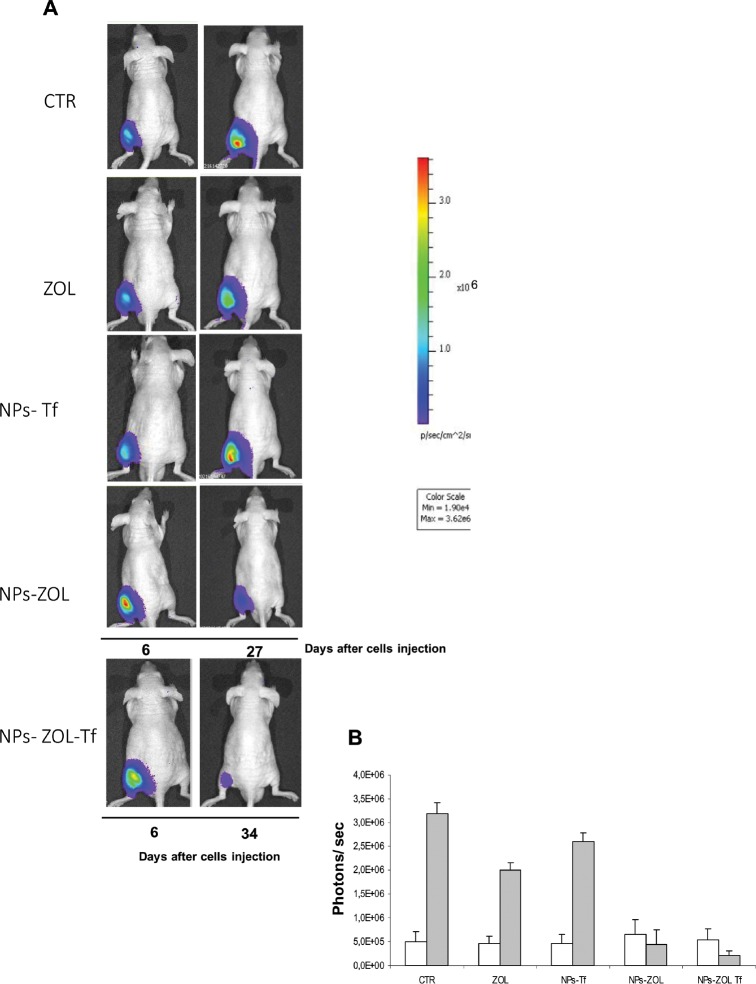
Antitumor effect of NPs-ZOL-Tf on luminescent xenograft A)Human GBM cells were transfected with a vector pcDNA3-luc containing the firefly luciferase gene. Real-time tumor growth of U373MG-LUC xenografts monitored by optical imaging as described in Materials and methods. Imaging was performed at baseline (day 6 after tumor cell injection) before administration of compounds and at day 27 or 34. Data were acquired and analyzed using the Living Image Software version 3.0 (Caliper Life Sciences). B) Histograms report bioluminescence in tumors from untreated or treated groups at day 6 (blank) and at day 27 (grey) after tumor cells injection. Error bars indicate ±SD.

After 6 days, when the tumor mass became palpable and visible by luminescence analysis, mice were divided into five groups: untreated mice, mice treated with free ZOL, with empty nanoparticles functionalized with transferrin, with NPs-ZOL and with NPs-ZOL-Tf. The growth curves of U373MG-LUC tumors treated with the different formulations are reported in figure 2. NPs-ZOL-Tf exhibited the highest antitumor efficacy. In fact, as summarized in table I, this treatment produced, at nadir of the effect, a significant (P= 0.009 vs untreated) tumor weight inhibition of about 41%, while the non-functionalized NPs-ZOL caused an about 31% tumour growth inhibition (P= 0.02) while free ZOL did not determine a particularly marked tumor growth inhibition (TWI 20%). The therapeutic activity of NPs-ZOL-Tf is also demonstrated by the significant (P= 0.03) delay of tumor growth (10 days) and by the increase of life survival of mice (23%). Interestingly, this treatment produced a complete tumor response in 1 out of six treated mice, while NPs-ZOL produce a stabilization of disease in 1 out of six treated mice. These data were also confirmed by the bioluminescence analysis of tumor growth during the treatment (Figure 3). Finally, it is interesting to note that all treatments were well tolerated by the animals, as no toxic deaths (Table I) or weight loss were recorded in animals.

### Effects on *intra-tissue* distribution of fluorescent NPs at confocal microscopy

With the aim to study the *intra-tissue* bio-distribution of NPs, we performed confocal microscopy analysis of tumor tissues collected at different times from mice intramuscularly bearing GBM tumours, injected with fluorescently-labeled-NPs. Mice were administered i.v. with a single dose of fluorescently labeled NPs-ZOL or NPs-ZOL-Tf and analysis was performed for content in cells from tumors at 6h, 12h and 24h after injection. Untreated control was assumed as negative control.

**Figure 4:**
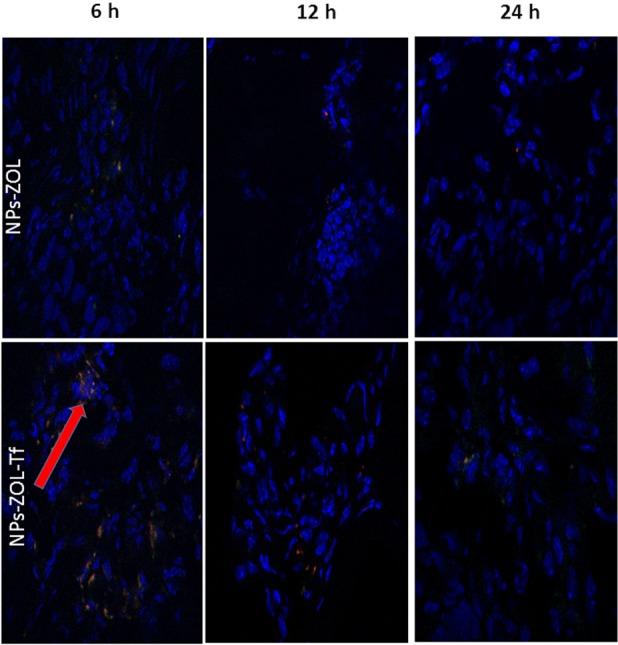
Intracellular distribution of fluorescent NPs at confocal microscopy Confocal microscopy images of tumor tissues collected at different times from mice, intramuscularly bearing GBM tumors, injected with fluorescently-labeled-NPs. Mice were randomized into 3 groups (vehicle, NP-ZOL and NP-ZOL-Tf) and i.v. administered with a single dose of fluorescently labeled NPs. In details, we analyzed the NP-ZOL and NP-ZOL-Tf content in cells from tumor at 6h, 12h and 24h after injection. Mice treated with vehicle were assumed as negative controls.

Results revealed that after 6h, NP-ZOL-Tf uptake was markedly increased as compared to negative control and NP-ZOL treated group. NPs accumulation decreased in a time-dependent manner after 12h (Figure 4). The quantitative representation of the results is shown in Supplementary Figure 1.

### Fluorescence microscopy intra brain

**Figure 5:**
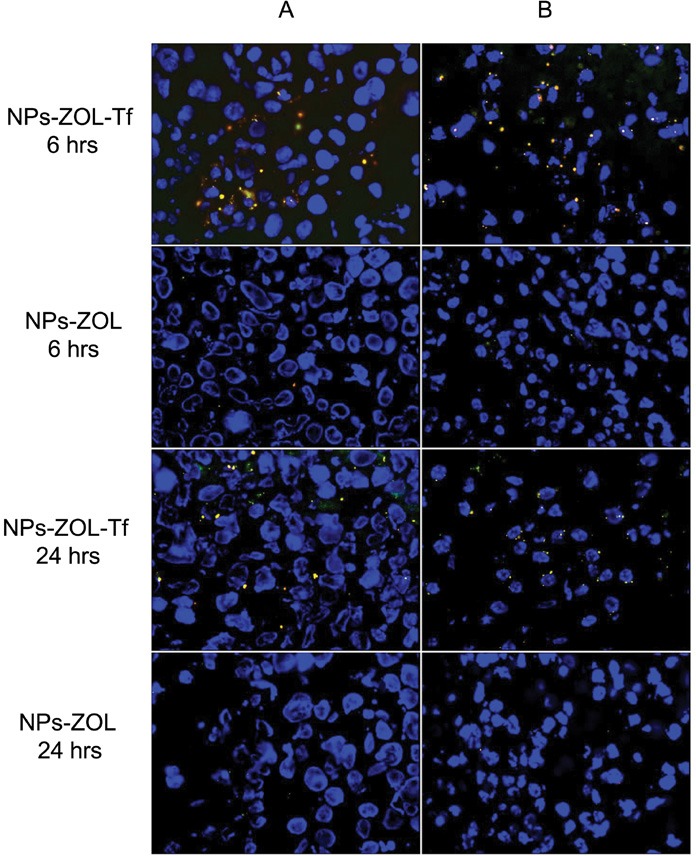
Distribution of NPs in mice brain by fluorescent microscopy Two weeks after the intra-cranially injection of U373-MG tumor cells (2.5 × 105 cells/mouse), the mice were treated with FITC/TRITC-double labelled NPs-ZOL and FITC/TRITC-double labelled NPs-ZOL-Tf, given i.v. at 20 μg/mouse. Brain sections from mice sacrificed 6 or 24 hrs after the treatment and examined by fluorescence microscope after nuclear DAPI counterstain, are shown. Original magnification, 400 X.

**Figure 6:**
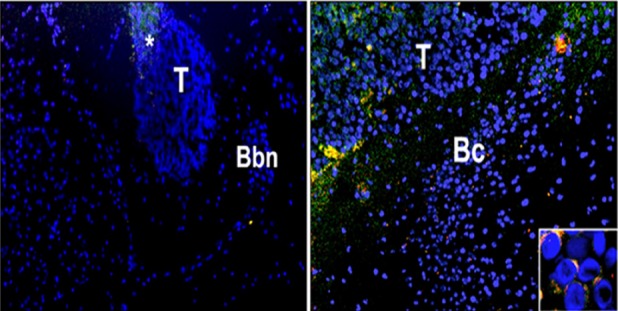
Effects on intrabrain distribution of NPs at fluorescence microscopy Distribution of NPs in brain samples from mice intra-cranially injected with the human glioblastoma cells U373-MG at 2.5 × 10^5^ cells mouse. Two weeks after tumor cells injection, the mice were treated with FITC/TRITC-double labelled NPs-ZOL, given i.v. at 20 μg/mouse. Frozen brain sections were examined by fluorescence microscope after nuclear DAPI counterstain. (A) Tumor (T) developed in the basal brain nuclei (Bbn) from a mouse sacrificed 6 hours after the first injection of FITC/TRITC-NPs-ZOL: accumulation of NPs-ZOL in the tumor mass is already evident. Original magnification, 100 X. (B) Tumor (T) developed in the brain cortex (Bc) from a mouse treated with FITC/TRITC-NPs-ZOL given three times a week for 3 consecutive weeks: most of the NPs-ZOL are present in the tumor mass but also some brain cortex cells appear to contain the NPs. Original magnification, 100 X. The insert shows the FITC/TRITC-NPs-ZOL uptake in the cytoplasm of tumor cells. Original magnification, 400 X.

To evaluate the ability of NPs formulation to cross the BBB and accumulate in the brain of mice bearing orthotopic tumors, mice were intra-cranially injected with U373MG-LUC and two weeks after tumor cell injection, treated with FITC/TRITC-double labelled NPs-ZOL or NPs-ZOL-Tf, given i.v. at 20 μg/mouse. Frozen brain sections were examined by fluorescence microscope after nuclear DAPI counterstain. The higher accumulation of NPs-ZOL-Tf than NPs-ZOL was evident in the tumor mass already 6 hours after the injection of FITC/TRITC-NPs-ZOL-Tf. The marked presence of NPs-ZOL-Tf in the brain was still observed 24 hours after the treatment, while the signal in the brain treated with NPs-ZOL was nearly undetectable (Figure 5 and Supplementary Figure 2). In particular, NPs-ZOL-Tf were evident in the tumor mass developed in the basal brain nuclei (Bbn) from a mouse sacrificed 6 hours after the first injection of FITC/TRITC-NPs-ZOL-Tf (Figure 6 A). After 3 consecutive weeks where FITC/TRITC-NPs-ZOL-Tf was given three times a week, most of the NPs-ZOL were present in the tumor mass developed in the brain cortex (Bc) but also some brain cortex cells appeared to contain the NPs (Figure 6 B). Moreover, the presence of a yellow fluorescence, due to the co-localization of the two dyes used to label NPs, suggests the presence of NPs still intact into the tissue (Figure 6).

### Antitumor efficacy of NPs-ZOL formulations against orthotopic GBM

**Figure 7:**
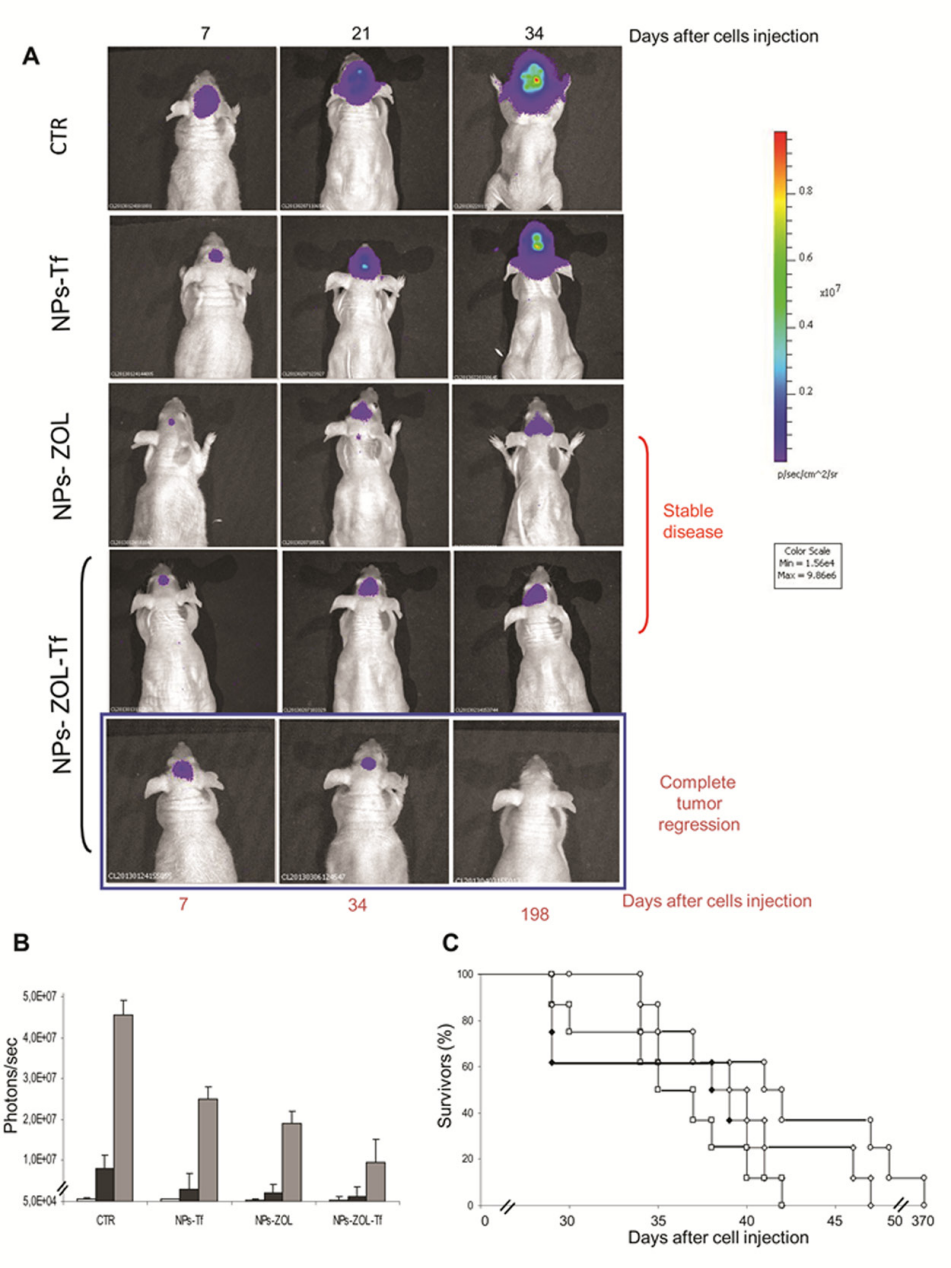
NPs- ZOL-Tf riduce the growth of U373 MG-LUC luminescent tumors in orthotopic xenografts Mice were anesthetized and injected intracranially with U373-MG LUC cells at 2,5 × 10^5^ cells/mouse, through the center-middle area of the frontal bone to a 2-mm depth, using a 0.1-mL glass microsyringe and a 27-gauge disposable needle. Mice were treated, starting at day 8 after cells injection, with NPs- Tf and NPs- ZOL-Tf at 20 μg/mouse for three times a week for 3 consecutive weeks. A)Tumor growth was monitored weekly using the IVIS imaging system 200 series (Caliper Life Sciences, Hopkinton, MA, USA). Briefly, mice were anesthetized with a combination of tiletamine–zolazepam (Telazol, Virbac, Carros, France) and xylazine (xylazine/Rompun BAYER) given intramuscularly at 2 mg/kg. The mice were injected intraperitoneally with 150 mg/kg D-luciferin (Caliper Life Sciences), and imaged in the supine position 10 min after luciferin injection. Representative tumor images on day 7 before administration of compounds and on day 21, 34 or 198 are shown. Data were acquired and analyzed using the Living Image Software version 3.0 (Caliper Life Sciences). B) Histograms report bioluminescence in tumors from untreated or treated groups at day 6 (blank), at day 27 (black) and at day 34 (grey) after tumor cells injection. Error bars indicate ±SD. C) Survival curves of the following groups (♦) untreated; (□) NPs- Tf; (◊) NPs-ZOL at 20 μg/mouse and (○) NPs- ZOL-Tf at 20 μg/mouse are reported.

Based on fluorescence microscopy analysis demonstrating the ability of both NPs-ZOL and NPs-ZOL-Tf to overcome the BBB and to accumulate in brain of mice bearing GBM tumours, we subsequently evaluated the therapeutic efficacy of these formulations. We inoculated intrabrain 2.5 x10^5^ U-373MG-LUC cells into immuno-compromised mice. This tumor model closely recapitulated histological phenotypes consistent with those of human GBM. After 8 days, when the tumor mass became visible by biolumincence analysis, the mice were divided into four groups: untreated mice, mice treated with empty NPs functionalized with Tf, with NPs-ZOL functionalized with Tf and mice treated with NPs-ZOL. As reported in Figure 7 and Table II, NPs-ZOL formulations were effective in limiting the growth of GBM and in particular, NPs-ZOL-Tf reduced tumor mass and cured some of treated animals. In fact, while all untreated or blank NPs-treated mice showed a progression of the disease, treatment with NPs-ZOL produced the stabilization of the disease in 2 out of eight mice treated. Interestingly, NPs-ZOL-Tf elicited a stronger antitumor effect determining the tumor mass stabilization in 2 out of eight mice and a decrease followed by the complete disappearance of the tumor in 1 out of eight mice (Figure 7 A). At that time, more than six months after the end of treatment, the mouse with complete tumor regression was still alive and in good conditions and bioluminescence analysis did not show the presence of tumor cells in mouse brain. After, tumor progressed in this mouse and the death was reported at day 370 after tumor cells injection (Fig. 7C). The higher activity of NPs-ZOL-Tf if compared to NPs-ZOL was also demonstrated by the increase of overall survival of mice (23 *vs* 13%, respectively), while treatment with blank NPs-Tf was ineffective.

**Table II:** Therapeutic efficacy of NPs- ZOL-TfU373MG-LUC on glioblastoma heterotopic xenografts

Treatment groups#	TWI* (%)	T-C § (days)	Stable diseaseΦ or complete response ΦΦ/mice treated	ILS& (%)	Body Weight Loss (%)♣	Lethal toxicity°
a) ZOL	20	5	0/6	10	0	0/6
b) NPs-ZOL	31	8	1/6Φ	10	0	0/6
c) blank NPs-Tf	14	2	0/6	0	0	0/6
d) NPs- ZOL-Tf	41	10	1/6ΦΦ	23	0	0/6

# U373-MG-LUC tumor bearing-mice were treated i.v. at 20 μg/mouse/d on days 6, 8, 10, 13, 15, 17, 20, 22, 24 after tumor cell injection.

* Tumor weight inhibition was calculated at the nadir of the effect. Statistical analysis: b vs untreated, p=0.02; d vs untreated, p= 0.009.

§ Calculated as the median times for treated (T) and control (C) tumors to reach the same size (1000 mg). Statistical analysis: b vs untreated, p= 0.03; d vs untreated, p= 0.038.

Φ Stable disease was defined as the maintenance for at least three weeks of the same tumor weight as the start of treatment and ΦΦ Complete response was defined as the disappearance of tumor as evaluated by palpability, for at least three weeks in the course of treatment.

& Increase in lifespan. ILS of treated mice was calculated compared their median survival time (MST) with those of untreated mice. Statistical analysis: d vs untreated, p=0.021.

° Number of toxic deaths/total number of treated mice.

Φ Body Weight Loss was defined as the percentage of loss of body weight during the treatment compared to body weight at the start of treatment.

Interestingly, NPs formulations were well tolerated as no toxic deaths or body weight loss was reported in animals (Table II and III).

**Table III:** Antitumor efficacy of NPs-ZOL-Tf on intracranial U373-MG-LUC glioblastoma

Treatment groups &	ILS% *	Tumor progression/mice treated #	Stable disease/mice treated #	Complete tumor regression/mice treated #	Body Weight Loss % $
a) blank NPs-Tf	6	8/8	0/8	0/8	16
b) NPs-ZOL	13	6/8	2/8	0/8	12
c) NPs-ZOL-Tf	23	4/8	3/8	1/8	8

& U373-MG-LUC tumor bearing-mice were treated i.v. with the different formulations at 20 μg/mouse/d on days 8, 10, 12, 15, 17, 19, 22, 24, 27 after tumor cell injection.

* Increase of life survival. Median survival time of control group was 34 days (29–42).

# Tumor progression was defined as the progressive increase of bioluminescent signal compared to the starting of treatment, stable disease as the observation of the same level of bioluminescent signal and complete tumor regression as an undetectable bioluminescent signal, lasting for at least three weeks.

$ Body Weight Loss was defined as the percentage of loss of body weight during the treatment compared to body weight at the start of treatment.

Overall, our results clearly demonstrate the therapeutic efficacy of NPs-ZOL-Tf against experimental models of human GBM, even on orthotopic tumours that closely mimic the clinical setting.

## DISCUSSION

Brain tumors are neoplasms orphan of efficacious medical treatments due to the presence of BBB that limits the access of anti-cancer agents, to the CNS. Thus, the development of formulations able to deliver drugs into the brain is becoming a promising option in order to control this type of malignancy.

In previous works we demonstrated that ZOL, a third generation aminobisphosphonate, can be transformed in a powerful anticancer agent, if administered with nanotechnology-based formulations [9, 22]. In this work, we investigated if this approach can be proposed also in the treatment of GBM. Nanotechnologies have been widely used for the targeted delivery of anticancer drugs [19, 23]. Brain tumors are generally characterized by vasogenic oedema with dramatic increase of the intracranial pressure, and consequent breakdown of the BBB, associated with down-regulation of tight junction [24]. In this condition, colloidal particles can access to the CNS thus bringing their drug cargo into the brain [17]. First experiments were performed *in vitro*, where we found that ZOL, only when encapsulated in the self-assembling NPs, has antiproliferative activity against U373 MG-LUC GBM cells. In the case of GBM, anti-cancer agents conventionally used in the clinic for the treatment of GBM are TMZ and FTM. In particular, TMZ, which represents the gold standard treatment in GBM, is currently used in combination with radiation therapy or alone after surgery and radiotherapy [1, 2]. On these bases, we evaluated the cytotoxic effects of the TMZ and FTM on U373 MG- LUC GBM cells. The cell line showed refractoriness *in vitro* to treatment with either TMZ or FTM. On the other hand, NP-ZOL was much more potent than TMZ and FTM in inducing growth inhibition of GBM cells. Active targeting has been also used to enhance drug delivery into the CNS. In particular, ligands for specific receptors have been used to promote NP access to the brain [25, 26]. Among them, Tf receptors are highly present on the endothelial cells of the BBB for the regulation of brain uptake of iron [27]. Moreover, Tf receptors are overexpressed in almost all cancers and, according to this, we found overexpression of Tf receptors also in U-373MG-LUC cells (data submitted for publication). Starting from these considerations, the previously developed self-assembling NPs were modified with Tf electrostatically bound to their surface in order to potentiate their delivery efficacy against GBM cells and to enhance crossing of the BBB. Thus, the following step of the work was to investigate if the presence of Tf in the formulation could affect the ZOL activity *in vitro*. Our results show that both NPs-ZOL and NP-ZOL-Tf induced a much more cytotoxic activity than standard cytotoxic agents in GBM cells. In particular, in clonogenic assay NP-ZOL-Tf resulted more effective in reducing the survival of cells comparing NPs-ZOL. This improved effect of NP-ZOL-Tf was not evident in MTT assay. This is not surprising when one considers that clonogenic assay is a long-term assay in which the ability of NPs-ZOL-Tf to increase the amount of drugs in cells, could be fully exploited. The increase of the effectiveness of ZOL-encapsulated into self-assembling NPs compared to free ZOL was also confirmed by the *in vivo* experiments. In particular, in a heterotopic model of GBM, NP-ZOL-Tf induced an about 41%, NP-ZOL an about 31% and free ZOL an about 20% inhibition of tumor growth, respectively. The greater effectiveness of NPs-ZOL-Tf if compared to NPs-ZOL *in vivo*, could be due to active targeting of tumor cells overexpressing the Tf receptors, that could also enhance the NP uptake into the cells [28]. Moreover, the presence of Tf on the NPs-ZOL-Tf may enhance accumulation into the tumor, by interaction with the Tf receptors expressed on the endothelial cells of the vessels irrorating the tissue [29]. This hypothesis seems to correlate with confocal microscopy analysis of tumor tissues excised by mice treated with fluorescent NPs. Indeed, confocal microscopy images showed that the presence of NPs in tumor tissues was significantly higher in mice treated with NPs-ZOL-Tf than in mice treated with NP-ZOL.

A major obstacle in the clinical management of GBM is the presence of BBB, which restricts the entrance of circulating therapeutics in the brain tissue. Therefore, we investigated the antitumor effect of the developed formulations in an orthotopic model of GBM. We found that NPs-ZOL significantly reduced the tumor growth with stabilization of the disease in 2 out of eight treated mice. This is not surprising because GBM itself can disrupt BBB integrity, through mechanisms such as secretion of soluble factors that actively degrade tight junctions [30], as well as formation of abnormal blood vessels with defective expression of tight junction proteins [31, 32]. NPs-ZOL can access to the brain taking advantage of the enhanced permeability of BBB. However, while large and advanced brain tumors exhibit especially disrupted BBB integrity, subregions with intact BBB are also present in areas of less advanced brain tumors, which hamper the uptake of drugs leading to cancer cell treatment resistance. Therefore, NPs modification with Tf, in order to target Tf receptor highly expressed in the BBB endothelial cells, should result in enhanced brain delivery of ZOL by transcytosis. Finally, the targeting of Tf-overexpressing GBM cells could lead to additional increase of ZOL antitumor activity on GBM. Therefore, to verify the validity of our hypothesis, we compared the efficacy of the NPs-ZOL and NPs-ZOL-Tf in an orthotopic model of GBM, in which the NPs have to overcome the BBB to access the tumor. Interestingly, the dual BBB and tumor targeting strategy resulted in a marked therapeutic efficacy of NPs-ZOL-Tf since, in mice treated with this formulation, a stabilization of the disease and a complete response with regression of tumor and cure in some cases were observed. These results were supported by the fluorescence images in which a significant fluorescence associated to the NPs was found only in the tumor tissues excised from animal treated with NPs-ZOL-Tf. Interestingly, especially after 6 h from the inoculation, the NPs were found especially into the tumor mass, probably, indicating effective tumor targeting. The co-localization of the two fluorescently dye, namely chol-BIODIPY and DHPE-CF, suggest that intact NP should arrive into the tumor. Further studies should be required to clarify the intracellular pathway of the Tf-modified or unmodified NPs, and the mechanism of ZOL release into the cytoplasm.

In conclusion, this manuscript provides evidence that the newly developed formulation NPs-ZOL-Tf, allows the successfully use of ZOL against GBM. Although we did not give conclusive evidence of the ZOL entry into the brain, in this work, we reported that this bisphosphonate, when encapsulated in self-assembling NPs have an antitumor activity on GBM. On the other hand, it is well known that ZOL when given free have no pharmacological effect in CNS [33]. The NPs modification with Tf allowed the achievement of enhanced antitumor activity in a heterotopic model of GBM. The advantage of Tf introduction into the formulation was more evident when investigating the effect of the NPs-ZOL-Tf in an orthotopic model of GBM, in which the drug has not only to reach the tumor, but also to cross the BBB. The results observed in this experimental animal model, namely the stabilization of the tumor in all the animal and complete regression in one out of eight, opens a new scenario in which it could have a strong impact for the treatment of brain tumors, for which a paucity of effective treatments exists.

## MATERIALS AND METHODS

### Ethics Statement

The procedures involving mice were in compliance with Regina Elena National Cancer Institute animal care guidelines and with national and international directives (D.L. March 4, 2014, no. 26; directive 2010/63/EU of the European parliament and of the council; Guide for the Care and Use of Laboratory Animals, United States National Research Council, 2011).

### Materials

Unless otherwise stated, all chemicals were from Sigma-Aldrich (Saint Louis, MO). 1,2-dioleoyl-3-trimethylammonium-propane chloride (DOTAP) and 1,2-diacyl-sn-glycero-3-phosphoethanolamine-N-[methoxy(polyethylene-glycol)-2000] (DSPE-PEG2000) were obtained from Lipoid GmbH (Cam, Switzerland). RPMI 1640 were purchased from FlowLaboratories (Milan, Italy). Tissue culture plasticware was from Becton Dickinson (Lincoln Park, NJ). ZOL was provided by Novartis (Novartis, Basilea, Svizzera). TMZ (Sigma Life Science) and FTM were provided by Prof. C. Leonetti (Experimental Chemotherapy Laboratory, Regina Elena National Cancer Institute IRCCS - Rome). NPs were provided by Prof. G. De Rosa (Department of Pharmaceutical Chemistry, University of Naples,‘‘Federico II’’).

### Preparation of ZOL-containing self-assembling NPs

In this study, we modified the surface of previously developed NPs-ZOL with human Tf. In detail, Tf modified NPs-ZOL were prepared as follows. In a first step, PEGylated cationic liposomes consisting of DOTAP/chol/DSPE-PEG_2000_ (1:1:0.5 weight ratio) were prepared by hydration of a thin lipid film followed by extrusion. The lipid mixture was dissolved in 1 ml of a mixture chloroform/methanol (2:1 v/v). The organic solution was removed by N_2_, and the film was further dried under vacuum. Then, the lipid film was hydrated with 1 ml of sterile water and the resulting dispersion was extruded using a thermobarrel extruder system. In a second step, pre-formed PEGylated cationic liposomes were mixed with human Tf (10 mg/ml in phosphate buffer at pH 8.0) at a volume ratio of 1:1, at room temperature for 15 min, obtaining the so-called Tf-PEGylated cationic liposomes. Separately, calcium phosphate nanoparticles (CaP NPs) containing ZOL were prepared as previously described by Salzano G et al., [22]. Briefly, an aqueous solution of calcium chloride (18 mM) was added, dropwise and under magnetic stirring, to an aqueous solution on dibasic hydrogen phosphate (10.8 mM). The pH of both solutions was adjusted beforehand to 9.5 with NaOH 0.1M. CaP NPs were obtained by filtration of the suspension through a 0.22 μm filter. The dispersion was then mixed with an aqueous solution of ZOL (50 mg/ml of ZOL in phosphate buffer at pH 9.5), resulting in CaP NPs containing ZOL (CaPZ NPs). Finally, NPs-ZOL-Tf were prepared by mixing Tf-PEGylated cationic liposomes complex with CaPZ NPs, at a volume ratio of 1:0.5, at room temperature for 15 min. Plain Tf-modified NPs (NPs-Tf), ZOL-encapsulating NP without Tf (NPs-ZOL) and plain NPs were prepared similarly. Each formulation was prepared in triplicate. Fluorescently labelled NPs were prepared similarly, by introducing Cholesteryl 4,4-Difluoro-5-(4-Methoxyphenyl)-4-Bora-3a,4a-Diaza-s-Indacene-3-Undecanoate (chol-BODIPY) (Life Technologies, USA) e 1,2-dioleoyl-sn-glycero-3-phosphoethanolamine-N-(carboxyfluorescein) ammonium salt (DHPE-CF) (Avanti Polar, USA) in the lipid mix, in the following ratio DOTAP/chol/DSPE-PEG2000/chol-BODIPY/DHPE-CF (1:1:0.5:0.025:0.025 weight ratio).

### Characterization of ZOL-containing self-assembling NPs

The mean diameter of NPs was determined at 20°C by photon correlation spectroscopy (PCS) (N5, Beckman Coulter, Miami, USA). Each sample was diluted in deionizer/filtered water (0.22 μm pore size, polycarbonate filters, MF-Millipore, Microglass Heim, Italy) and analyzed with detector at 90° angle. As measure of the particle size distribution, polydispersity index (P.I.) was used. For each batch, mean diameter and size distribution were the mean of three measures. For each formulation, the mean diameter and P.I. were calculated as the mean of three different batches. The zeta-potential (ζ) of the NPs surface was measured in water by means of a Zetasizer Nano Z (Malvern, UK). Data of ζ were collected as the average of 20 measurements. ZOL analysis was carried out by reverse phase high performance liquid chromatography (RP-HPLC) as previously reported [22]. The incorporation efficiency of ZOL in NPs-ZOL-Tf was determined as follows: 1 ml of NPs dispersion was ultracentrifuged (Optima Max E, Beckman Coulter, USA) at 80.000 rpm at 4°C for 40 min. Supernatant was carefully removed and analyzed to determine un-incorporated ZOL concentration by RP-HPLC. The results have been expressed as complexation efficiency, calculated as the ratio between the amount of ZOL present in the supernatant and the amount of ZOL theoretical loaded.

### Cell lines and cytotoxic assay

Human U373MG GBM cells were transfected with pcDNA3-luc (U373MG-LUC) as previously described [34] and were grown in RPMI medium in a humidified incubator containing 5% CO2 at 37°C. After trypsinization, the cells were plated in 100μL of medium in 96-well plates at a density of 2 × 10^3^ per well. One day later, the cells were treated with free ZOL, NPs-ZOL functionalized or not with transferrin, TMZ and FTM at increasing concentrations. Cell proliferation was evaluated by MTT as previously described [35]. In clonogenic assay, the cells were seeded at 5 × 10^4^ cells/plate and exposed 24 hrs later to TMZ and FTM for 48 hrs and to NPs-ZOL and NPs-ZOL-Tf for 72 hrs. Cell survival was evaluated as previously described [36].

### *In vivo* experiments

CD-1 male nude (nu/nu) mice, 6–8 weeks old and weighing 22–24 g were purchased from Charles River Laboratories (Calco, Italy). For heterotopic experiments, immunosuppressed mice were injected intramuscularly (i.m.) into the hind leg muscles of mice at 3×10^6^ U373MG-LUC GBM cells /mouse. After 6 days (when a tumor mass of about 300mg was evident) mice were randomized, divided in five groups and treatment started. The following groups were evaluated: untreated; free ZOL; empty NPs plus transferrin (NPs-Tf); ZOL-encapsulating NPs (NPs-ZOL) and ZOL-encapsulating NPs plus transferrin (NPs-ZOL-Tf). Mice were treated intravenously (i.v.) with NPs or with 20 μg of free ZOL, NPs-ZOL or NPs-ZOL- Tf for three times a week for 3 consecutive weeks. This scheduling of treatment was chosen based on previous published experiments [16].

Tumor sizes were measured three times a week in two dimensions by a caliper and tumor weight was calculated using the following formula: a×b2/2, where a and b are the long and short sizes of the tumor, respectively. Antitumor efficacy of treatments was assessed by the following end-points: a) percent tumor weight inhibition (TWI%); b) tumor growth delay, evaluated as T - C, where T and C are the median times for treated and control tumors, respectively, to achieve equivalent size; c) complete tumor regression, defined as tumor disappearance, as evaluated by palpability, lasting for at least 10 days during or after treatment period; d) increase of mice survival by euthanizing the animals, for ethical reasons, when the tumors reached 3 g in weight; e) stable disease, defined as the maintenance for at least three weeks of the same tumor weight as the start of treatment; f) complete response, defined as the disappearance of tumor, for at least three weeks in the course of treatment. Each experimental group included six mice and experiments were repeated at least twice.

For orthotopic experiments, mice were anesthetized with a combination of tiletamine–zolazepam (Telazol, Virbac, Carros, France) and xylazine (xylazine/Rompun BAYER) given intramuscularly at 2 mg/kg and injected intracranially with U373MG-LUC cells at 2.5 × 10^5^ cells/mouse, through the center-middle area of the frontal bone to a 2-mm depth, using a 0.1-mL glass microsyringe and a 27-gauge disposable needle. One hour prior to intracranial implantation, the mice were weighed and pre-medicated with an orally administration of 0.5 mg/kg/d of Metacam (meloxicam) in saline to control for post-operative pain and inflammation. The medication was carried out until the end of the experiment. Animals were closely monitored by visual inspection and weighed daily from start of treatment and sacrificed when signs of tumor burden (especially weight loss >20% and severe neurological dysfunction) were evident. The time to this moment since GBM cells injection is considered as ‘survival time’. Mice were treated i.v., starting at day 8 after cells injection, with Blank NPs-Tf, NPs-ZOL and NPs-ZOL-Tf at 20 μg/mouse for three time a week for 3 consecutive weeks. Experiments with ZOL given free were not performed due to the well-known inability of this drug to cross the BBB [33]. Each experimental group included eight mice and experiments were repeated twice.

### Bioluminescence imaging analysis

Mice bearing i.m. or intra brain U373MG-LUC tumors were imaged using the IVIS imaging system 200 series (Caliper Life Sciences, Hopkinton, MA, USA). Briefly, mice were anesthetized with a combination of tiletamine–zolazepam (Telazol, Virbac, Carros, France) and xylazine (xylazine/Rompun BAYER) given intramuscularly at 2 mg/kg. Then mice were injected intraperitoneally with 150 mg/kg D-luciferin (Caliper Life Sciences), and imaged in the supine position 10 min after luciferin injection. Imaging was performed at baseline before the start compound administration and several times during the experiment. Data were acquired and analyzed using the living image software version 3.0 (Caliper Life Sciences).

### Analysis of intratissue distribution of fluorescent NPs at confocal microscopy

Mice were injected i.m. with 3 × 10^6^ U373 MG-LUC cells/mouse and treated with fluorescent NPs-ZOL and NPs-ZOL-Tf for 6, 12 and 24h. At the end of the experiment, mice were sacrificed and tumors were excised. Frozen sections of tumor tissues were cut with a cryostat on a slide. Tissues were fixed for 20 minutes with a solution at 3% of paraformaldehyde (PFA) and permeabilized for 10 minutes with 0.1% Triton X-100 in phosphate-buffered saline (PBS) at room temperature. After several washes the slides were mounted on microscope slides by DAPI. The analyses were performed with a Zeiss LSM 510 microscope equipped with a plan-apochromat objective × 63 (NA 1.4) in oil immersion. The fluorescence of the Alexa 488 and Alexa 633 was collected in multi-track mode using as emission filters a BP505-530 and LP650 a respectively.

### Intra brain fluorescence microscopy

To evaluate the ability of NPs to overcome blood brain barrier and accumulate in orthotopic GBM, mice bearing intracranial U373 MG-LUC tumor were treated with FITC/TRITC-double labelled NPs-ZOL or NPs-ZOL-Tf, given i.v. at 20 μg/mouse and then sacrificed 6 and 24 hours after the treatment. Whole brains of untreated and FITC/TRITC-double labelled NPs-ZOL or NPs-ZOL-Tf treated mice were embedded in OCT (Bayer) and frozen in liquid nitrogen. 5μm frozen sections, taken at different brain zone, were harvested on glass slides, counterstained with DAPI and examined by epifluorescence microscopy (Nikon Diaphot TMD; Nikon, Mellville, New York)

### Statistical analysis

The Student's t-test (unpaired, two-tailed) was used for comparing statistical differences. Survival curves of mice were generated by Kaplan–Maier product-limit estimate, and statistical differences between the various groups were evaluated by log-rank analysis with Yates correction (software Primer of Biostatistics, McGraw-Hill, New York, NY, USA). Differences were considered statistically significant when P< 0.05.

## SUPPLEMENTARY MATERIAL FIGURES


