# Supplementary material for: Medical treatment of orthotopic glioblastoma with transferrin-conjugated nanoparticles encapsulating zoledronic acid
Source: Oncotarget. 2014 Jul 9;5(21):10446–59. doi: 10.18632/oncotarget.2182 (PMC4279385; doi:10.18632/oncotarget.2182)
Supplement: Supplementary file 1 [file oncotarget-05-10446-s001.pdf]

Medical treatment of orthotopic glioblastoma with transferrin-conjugated nanoparticles encapsulating zoledronic acid

Supplementary Material

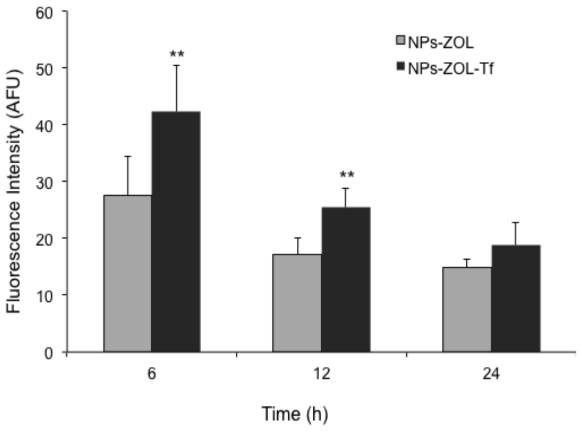

Suppl Figure 1: **Fluorescence intensity of the intracellular NPs.** The fluorescence intensity was calculated with Image J software analysis and expressed as arbitrary fluorescence units (AFU). The mean (n=5) of the unspecific fluorescence of the secondary antibodies was calculated by Image J software analysis and subtracted. Data are expressed as mean±SD with **P <0.05 vs. NPs-ZOL.

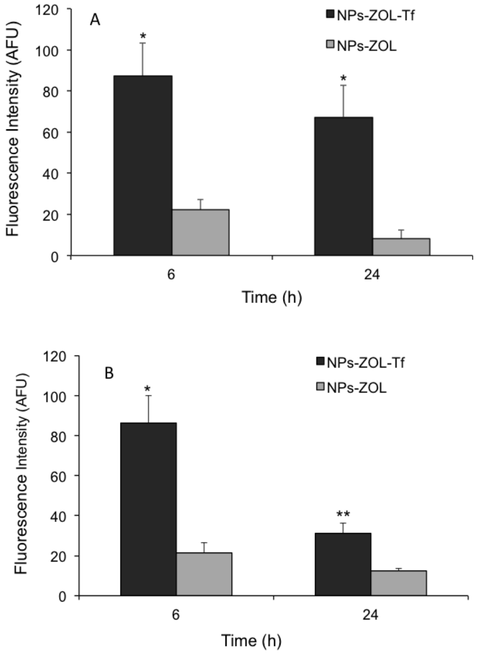

Suppl Figure 2: Fluorescence intensity of NPs distribution in mice brain. The fluorescence intensity of the FITC/TRITC-double labeled NPs-ZOL and FITC/TRITC-double labeled NPs-ZOL-Tf in mice brain specimens from tumor mass at 6 hrs (A) and 24 hrs (B) after the beginning of the administration of the different agents in the same mouse was calculated by Image J software analysis and expressed as arbitrary fluorescence units (AFU). Data are expressed as mean±SD with *P <0.01 or **P <0.05 vs. NPs-ZOL.
